# Flexible Bronchoscopy with Multiple Modalities for Foreign Body Removal in Adults

**DOI:** 10.1371/journal.pone.0118993

**Published:** 2015-03-13

**Authors:** Yueh-Fu Fang, Meng-Heng Hsieh, Fu-Tsai Chung, Yao-Kuang Huang, Guan-Yuan Chen, Shu-Min Lin, Horng-Chyuan Lin, Chin-Hwa Wang, Han-Pin Kuo

**Affiliations:** 1 Department of Thoracic Medicine, Chang Gung Foundation, Chang Gung Memorial Hospital, Taoyuan, Taiwan; 2 College of Medicine, Chang Gung University, Taoyuan, Taiwan; 3 Division of Thoracic and Cardiovascular Surgery, Chang Gung Memorial Hospital, Chia-Yi, Taiwan; Oregon Health and Science University, UNITED STATES

## Abstract

**Objectives:**

Aspiration of the lower airways due to foreign body is rare in adults. This study aimed to determine the outcome of patients who received flexible bronchoscopy with different modalities for foreign body removal in the lower airways.

**Patients and Methods:**

Between January 2003 and January 2014, 94 patients diagnosed with foreign body in the lower airways underwent flexible bronchoscopy with different modalities, which included forceps, loop, basket, knife, electromagnet, and cryotherapy. The clinical presentation, foreign body location and characteristics, and applications of flexible bronchoscopy were analyzed.

**Results:**

Forty (43%) patients had acute aspiration, which developed within one week of foreign body entry and 54 (57%) had chronic aspiration. The most common foreign bodies were teeth or bone. More patients with chronic aspiration than those with acute aspiration were referred from the out-patient clinic (48% vs. 28%), but more patients with acute aspiration were referred from the emergency room (35% vs. 6%) and intensive care unit (18% vs. 2%). Flexible bronchoscopy with different modalities was used to remove the foreign bodies (85/94, 90%). Electromagnet or cryotherapy was used in nine patients to eliminate the surrounding granulation tissue before foreign body removal. In the nine patients with failed flexible bronchoscopy, eight underwent rigid bronchoscopy instead and one had right lower lung lobectomy for lung abscess.

**Conclusions:**

Flexible bronchoscopy with multiple modalities is effective for diagnosing and removing foreign bodies in the lower respiratory airways in adults, with a high success rate (90%) and no difference between acute and chronic aspirations.

## Introduction

Foreign body aspiration into the lower airway is less likely in adults than in children.(1–8) Some adult patients have acute aspiration within one week but often have no acute symptoms that occur in children or infants. Others have no acute aspiration history within one month but may have only chronic cough without dyspnea, wheeze, or chest pain. Thus, the diagnoses of aspirated foreign bodies may be delayed.(1, 2) In such patients, foreign bodies are incidental findings during bronchoscopy for lung collapse or delayed pneumonia resolution. Foreign bodies may be embedded in granulation tissue and difficult to remove.(2, 9)

Flexible or rigid bronchoscopy is the method used to diagnose and remove foreign bodies. Flexible bronchoscopy is more convenient as patients are only lightly sedated. Granulation tissue may grow and cover the foreign body in patients with chronic aspiration. As such, the foreign body may be hard to remove if only suction, forceps, loops, or baskets are used. In therapeutic bronchoscopy or cryobiopsy, electromagnet or cryotherapy is applied.(10–14) These methods can be performed in flexible bronchoscopy for patients with foreign body and granulation tissue. The electromagnet or cryotherapy can cut or destroy the granulation tissue before the forceps, loops or baskets are used to remove the foreign body.

This study reviewed the records of patients who received bronchoscopy between January 2003 and January 2014 for foreign body in the lower airways to determine their outcomes and the success rate of the procedure for removing foreign bodies.

## Patients and Methods

All patients who received flexible bronchoscopy at the Interventional Bronchoscopy Center of Chang Gung Memorial Hospital, Linkou Medical Center between January 2003 and January 2014 were initially included. The patients were referred from the outpatient clinic, emergency room, general ward, and intensive care unit. Flexible bronchoscopy was the first method used, within 24 hours or immediately at the emergency room, to diagnose the presence of a foreign body in the lower airways in adult patients in the hospital. Based on the hospital protocol, the foreign body was removed in the same bronchoscopic examination used for diagnosing the foreign body in the lower airway.

Patients diagnosed with foreign body in their lower respiratory tract were selected. They all received flexible bronchoscopy as diagnostic and therapeutic management. The modalities used included forceps, loop, basket, knife, electromagnet, and cryotherapy. Electromagnet, electro-forceps, Nd-YAG, or cryotherapy was used to destroy or remove granulation tissue. In some patients, endobronchial ultrasound was used to detect foreign bodies embedded in the granulation tissue.

The patients’ general characteristics, indications of bronchoscopy, foreign body location, types of foreign bodies, and modality used for removing the foreign bodies were assessed. Acute aspiration was defined as aspiration that developed within one week of foreign body entry while chronic aspiration was defined as chocking history more than one month or no definite chocking history. Asymptomatic patients with abnormal X-ray results, including those with incidental findings of suspected foreign bodies in the airways, lung collapse, or delayed resolution of pneumonia were considered as chronic aspiration. Differences between the acute and chronic aspiration groups, including the locations and types of foreign bodies, use of electromagnet or cryotherapy, and success rate were analyzed.

### Ethics statement

Our study was a retrospective study of chart review. All the patients had signed the permits of interventional bronchoscopy, included removing foreign body, and electrocoagulation or cryotherapy for granulation tissue. The permits of all procedures were reviewed by the institutional Review Board of the Chang Gung Medical Foundation. The additional informed consents were not required for this retrospective study of chart review. The identified information of the patients, included their names and chart numbers, was deleted for de-identification before data analysis. The method assurance of patient confidentiality, and design of project were all approved by the institutional Review Board of the Chang Gung Medical Foundation (IRB No. 100–3211B)

## Results

### Clinical Characteristics

After reviewing 38314 patients who received flexible bronchoscopy, 94 were diagnosed with foreign bodies in their lower respiratory tract, including 67 (71%) males and 27 (29%) females. (Table 1) Thirty-seven (39%) were referred from the out-patient clinic, 17 (18%) from the emergency room, 32 (34%) from the general ward, and eight (9%) from the intensive care unit. Fifty-one (54%) patients had a definite history of acute aspiration or chest x-rays that showed suspected a foreign body in the lower airways, while 43 (46%) had no definite history of aspiration or visible foreign body on their chest X-ray or computed tomography (CT) imaging.

**Table 1:** Patient Characteristics.

Sex		
Male	67	71%
Female	27	29%
Age range (median),years	18–98 (66)	
Patient source		
Outpatient clinic	37	39%
Emergency room	17	18%
Ward	32	34%
Intensive Care Unit	8	9%
Indications of bronchoscopy		
Chocking and/or visible foreign body in image	51	54%
Lung collapse or delayed resolution	43	46%
Location		
Trachea	4	4%
Right lung	55	59%
Right main bronchus	4 (4%)	
Right intermediate bronchus	21(22%)	
Right middle bronchus	6 (6%)	
Right lower bronchus	24(26%)	
Left lung	35	37%
Left main bronchus	20(21%)	
Left upper bronchus	5 (5%)	
Left lower bronchus	10(11%)	
Foreign body		
Tooth (original or artificial)	20	23%
Bone (chicken, fish, pork)	29	31%
Teeth stick	2	2%
Bean/corn/vegetable	17	18%
Shrimp	2	2%
Fiber/cotton	6	6%
Plump	4	4%
Peanut	4	4%
Mental wire	2	2%
Others	8	9%

### Removal of Foreign Body

**Fig 1:**
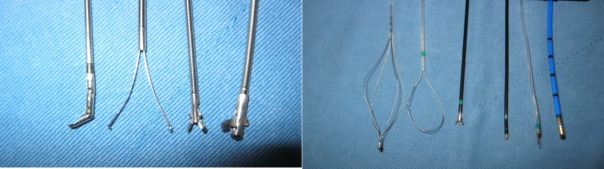
The multiple modalities of the flexible bronchoscopy included forceps, basket, loop, coagulation forceps, coagulation knife, and probe for cryotherapy.

In the 94 patients, four (4%) had a foreign body in the trachea, 55 (59%) had it in the right lung, and 35 (37%) had it in the left lung (Table 1). Twenty-nine (31%) had bone as the foreign body and 20 (23%) had a tooth. The other foreign bodies included vegetables, flosser, cotton, shrimp, core of plum, peanut, and metal wire. The modalities used for removing the foreign bodies were direct suction by bronchoscopy, forceps, loops, knife, basket, electromagnet, and cryotherapy (Fig. 1).

Foreign body removal was successful in 85 (90%) patients but failed in nine (10%). Eight of the nine patients received rigid bronchoscopy for removal of the foreign body, while one had right lung lobectomy for lung abscess. Nine (10%) of the 94 patients used electromagnet or cryotherapy to remove the granulation tissue that covered the foreign body.

### Acute and Chronic Aspiration

Forty (47%) patients were referred for acute aspiration, which occurred within one week, while 54 (53%) had chronic aspiration. (Table 2) More patients with acute aspiration than those with chronic aspiration were referred from the emergency room (14 [35%] vs. 3 [6%]) and intensive care unit (7 [18%] vs. 1 [2%]). On the other hand, more patients with chronic aspiration were referred from the out-patient clinic (26 [48%] vs. 11 [28%]) and general ward (24 [44%] vs. 8 [20%]).

**Table 2:** Comparison of Patients with Acute and Chronic Aspiration.

	Acute aspiration	Chronic aspiration	*p* value^#^
Number of patients	40	54	
Sex			
Male	28	29	0.11
Female	12	25	
Age range (median), years	20–98 (67)	18–83(63)	
Patient source			<0.001
Outpatient clinic	11	26	
Emergency room	14	3	
Ward	8	24	
Intensive Care Unit	7	1	
Location			0.90
Trachea	2	2	
Right lung	24	31	
Left lung	14	21	
Foreign body			<0.001
Tooth (original or artificial)	18	2	
Bone (chicken, fish, pork)	5	24	
Teeth stick	1	1	
Bean/corn/vegetable	4	13	
Shrimp	2	0	
Fiber/cotton	3	3	
Plump	2	2	
Peanut	1	3	
Mental wire	1	1	
Others	3	5	
Granulations	6	37	
Electromagnet or cryotherapy	1	8	0.049
Removal of foreign body			
Success	36	49	0.90
Failed	4	5	

^#^Fisher’s exact tests.

In acute aspiration, most patients had chocking of tooth (18/40, 45%). In patients with chronic aspiration, the most common foreign bodies were bone (24/54, 44%) or vegetables (13/54, 24%). Eight (15%) of fifty-four patients with chronic aspiration needed electromagnet or cryotherapy, whereas only one patient with acute aspiration needed electromagnet for foreign body removal. The success rate of foreign body removal was not different between the two groups (36 [90%] vs. 49 [91%]).

### Electromagnet and Cryotherapy

**Fig 2:**
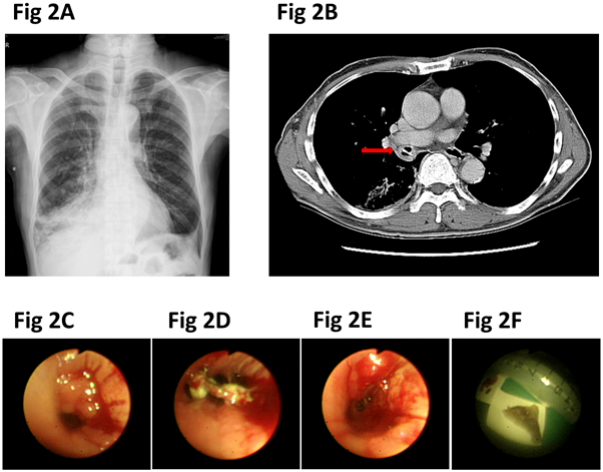
**(A)** Atelectasis of the right lower lung. **(B)** Foreign body in the right intermediate bronchus. **(C)** Granulation tissue covered the foreign body. **(D)** Bony foreign body after removing the granulation tissue by cryotherapy and forceps. **(E)** A patent right intermediate bronchus is noted after foreign body removal. **(F)** The foreign body.

Forty-three patients had granulation tissue, including nine who needed electromagnet or cryotherapy to remove the granulation tissue that partially or totally covered the foreign bodies (Fig. 2A and 2B). Electromagnet or cryotherapy was done for the granulation tissue or masses in the lower airway (Fig. 2C). The foreign body was removed after removal of the granulation tissue (Fig. 2D), which revealed a patent bronchial lumen (Fig. 2E and 2F).

### Failed Foreign Body Removal by Flexible Bronchoscopy

Nine patients had failed foreign body removal by flexible bronchoscopy. Four patients with acute aspiration received rigid bronchoscopy to remove the foreign body. One patient had garlic in the right intermediate bronchus and one had of plum in the right main bronchus with lung collapse. These two patients had complete airway obstruction. The other two patients had a prosthetic tooth in the right intermediate bronchus or the left main bronchus, with mucosal invasion and bleeding.

In the other five patients with chronic aspiration, two had a bone in the right lower lung bronchus, one had bone in the right intermediate bronchus, one had fish bone in the left main bronchus, and one had a catheter in the right intermediate bronchus. The one patient with a bone in the right lower lung bronchus had lung abscess that eventually needed lobectomy. The other four cases had foreign bodies that were deeply embedded in granulation tissue. There was easy bleeding whenever the bronchoscope passed the mucosa, making removal of the foreign bodies more difficult. These four patients received rigid bronchoscopy instead.

## Discussion

This study demonstrates a high prevalence of chronic aspiration secondary to a foreign body in adult patients. Lung collapse and obstructive pneumonia are common complications and the most common foreign bodies are bone in chronic aspiration and teeth in acute aspiration. There is also a predominance of male patients and involvement of the right bronchus. These results are similar to those of previous reports.(1, 2, 8)

About half of the patients have no history of acute aspiration or a visible foreign body in chest imaging. The manifestations are different in adults and in children, (1–3, 5, 7, 8)although some reports of adult patients also show similar results.(1, 2) This is due to the larger diameter of the adult bronchus, which cannot be totally obstructed by a foreign body. Some adult patients are asymptomatic, causing delayed diagnoses, such that these patients are diagnosed only after lung collapse or the development of obstructive pneumonia.

The prevalence of acute aspiration is higher in this study than in the report of Chen from another medical center in Taiwan.(2) In the current study setting, the Interventional Bronchoscopy Center can perform bronchoscopy for acute aspiration within 24 hours and immediately at the emergency room for patients with respiratory distress or respiratory failure. Thus, many patients with acute aspiration are referred to the emergency room and any special situation may increase the patient numbers with acute aspiration.

Of the eight patients in the intensive care unit, five had original tooth or artificial dentures in their lower airway. Two had aspiration of false teeth before intubation and two had aspiration of original teeth after intubation. Two had cotton aspiration and one had aspiration of shrimp. These patients received bronchoscopy under ventilator support and their foreign bodies were all removed by flexible bronchoscopy with different modalities. In the five patients with teeth in their lower airway, the foreign body was removed by bronchoscopy and the endotracheal tube withdrawn at the same time. The endotracheal tube was changed by bronchoscopic guidance after retrieval of the foreign body.

In the acute aspiration group, 18 (45%) patients have aspiration of teeth. This is because most patients can easily find their lost original or prosthetic teeth via chest X-ray or CT imaging. In contrast, the most common foreign bodies among chronic aspiration patients are bone and vegetable. This may be due to the different food preparations and habits of eating Chinese food.(1, 2, 6, 15, 16) In preparing Chinese food, people cook whole fish and like to eat small pieces of fish meat around fish bone. People also like to mixed meat and vegetables in soup or in the dinner plate. Bone or vegetables can easily enter the lower airways while eating soup. In Chinese food, people often take rice or noodles mixed with meat or vegetables. As the patient chews the rice or noodles, bone or vegetable fragments can roll into their lower airways. People may have no other symptoms except acute coughing as the foreign body enters their lower airways so they will not seek any diagnostic procedure to find the acute aspiration. They will be diagnosed as chronic aspiration when they have lung collapse or obstructive pneumonia.

In this study, the success rate of the one-step bronchoscopy to diagnose and remove a foreign body in the lower airways is high (85/94, 90%) and does not differ between acute and chronic aspiration patients (90% vs. 91%). This may result from early intervention and the multiple modalities of bronchoscopy. Early intervention can lead to the immediate removal of the foreign body, preventing more granulation formation. Of the 54 chronic aspiration patients, 37 (69%) had granulation, including eight (15%) who needed electromagnet or cryotherapy to eliminate the granulations before foreign body removal. Electromagnet and cryotherapy can increase the success rate in chronic aspiration patients. Four acute aspiration patients and four chronic aspiration patients had easy bleeding of the granulations that completely covered the foreign bodies. The foreign body was not removed by flexible bronchoscopy but by rigid bronchoscopy. It would be helpful to physicians to determine the number of times more than one or 2 more modalities required to manage airway foreign body. However, all foreign bodies in current study were successfully retrieved in one time procedure of flexible bronchoscopy. Difficult cases those could not be retrieved in one procedure would receive surgical management by surgeon. But the cases required surgical management were few (the numbers in the past 10 years).

Light sedation is commonly used for bronchoscopy to finish the procedure as soon as possible. However, in recent years, more patients receive bronchoscopy with moderate to deep sedation for less irritable movements and longer time to perform electromagnet and cryotherapy.

In Taiwan, people believe that fish could help patients’ recovery of health from their diseases. Most patients may eat fish when they suffered from some diseases. Especially, people in Taiwan like to eat “Milkfish” which is full of fish bone. Therefore, it is not uncommon that patients mis-swallow fish bone in Taiwan, which may cause airway aspiration, throat, or esophagus injury by fish bone! Such food like fish bone is usually not visible on chest X ray film.

There are multiple other case series in literature that highlight the same facts. This study is not novel and is descriptive. These are its major limitations! However, this current study is a cohort data during 10 years which included a large number of cases. In addition, we provided the different methods and tools to retrieve the foreign body such as forceps, basket, loop, coagulation forceps, coagulation knife, and probe for cryotherapy! We believed that this study would help the physicians to manage airway foreign body! Current definition of chronic aspiration in our study was defined as chocking history more than one month or no definite chocking history. This may not be adequate. However, We had searched references from midline, there were no any clear definitions of chronic aspiration.

In conclusion, flexible bronchoscopy with multiple modalities is effective for diagnosing and removing foreign bodies in the lower respiratory airways in adults, with a high success rate and no difference between acute and chronic aspirations.

### Conclusion

The most common foreign bodies in acute or chronic aspiration were teeth or bone in the lower airway of adult patients. Granulations were found in most patients with chronic aspiration and some foreign bodies were embedded in granulaton tissue. Flexible bronchoscopy with multiple modalities is a useful method to diagnose and remove the foreign bodies in these patients. The successful rate was high (90%) and no difference between acute and chronic aspiration as we applied multiple modalities, included electromagnet and cryotherapy, in these patients.
